# Diagnosis of Disease, &c.

**Published:** 1881-06

**Authors:** 


### BOOK NOTICES AND REVIEWS.

A GUIDE TO THE CLINICAL EXAMINATION OF PATIENTS AND THE DIAGNOSIS OF DISEASE. By Richard Hagan, M.D. Privat docent to the University of Leipzig. Translated from the Second Revised and Enlarged Edition, by G. E. Gramm, M.D., pp. 223, 12mo. Cloth. Price, $1.25. Boericke & Tafel, New York and Philadelphia, 1881.

This neat little volume contains all the most reliable points in diagnosis, condensed into a book that may be carried in the pocket. It makes an excellent elementary text-book for the student, and enables him the better to use the larger standard works on diagnosis. In its arrangement there is some slight suggestion of the tables for analysis of plants or for recognizing minerals. Of course, the subject will not admit of such plan in full; hence the present work is only a suggestion of such a method. The directions for auscultation, percussion, and for urinary analysis are exceedingly good, especially when we take into consideration the small size of the book. It is gotten up in excellent style, with good paper and clear type, so that it is a pleasure to read it. It must ultimately become a handy book of reference for every intelligent practitioner of medicine. W. M. J.

